# Creating the ICU of the future: patient-centred design to optimise recovery

**DOI:** 10.1186/s13054-023-04685-2

**Published:** 2023-10-21

**Authors:** Oystein Tronstad, Dylan Flaws, Sue Patterson, Robert Holdsworth, John F. Fraser

**Affiliations:** 1 Critical Care Research Group, The Prince Charles Hospital, Level 3 Clinical Sciences Building, Chermside, QLD 4032 Australia; 2 Faculty of Medicine, University of Queensland, Brisbane, QLD Australia; 3 Physiotherapy Department, The Prince Charles Hospital, Brisbane, QLD Australia; 4 Department of Mental Health, Metro North Mental Health, Caboolture Hospital, Caboolture, QLD Australia; 5 School of Clinical Sciences, Queensland University of Technology, Brisbane, QLD Australia; 6 School of Dentistry, University of Queensland, Brisbane, QLD Australia

**Keywords:** Acoustics, Environment, ICU, Light, Noise, Redesign, Technology

## Abstract

**Background:**

Intensive Care survival continues to improve, and the number of ICU services is increasing globally. However, there is a growing awareness of the detrimental impact of the ICU environment on patients, families, and staff. Excessive noise and suboptimal lighting especially have been shown to adversely impact physical and mental recovery during and after an ICU admission. Current ICU designs have not kept up with advances in medical technology and models of care, and there is no current ‘gold-standard’ ICU design. Improvements in ICU designs are needed to optimise care delivery and patient outcomes.

**Methods:**

This manuscript describes a mixed-methods, multi-staged participatory design project aimed at redesigning and implementing two innovative ICU bedspaces. Guided by the action effect method and the consolidated framework for implementation research, the manuscript describes the processes taken to ensure the patient-centred problems were properly understood, the steps taken to develop and integrate solutions to identified problems, and the process of implementation planning and rebuilding in a live ICU.

**Results:**

Two innovative ICU bedspaces were rebuilt and implemented. They feature solutions to address all identified problems, including noise reduction, optimisation of lighting, access to nature via digital solutions, and patient connectivity and engagement, with solutions developed from various specialty fields, including IT improvements, technological innovations, and design and architectural solutions. Early evaluation demonstrates an improved lighting and acoustic environment.

**Conclusions:**

Optimising the ICU bedspace environment and improving the lighting and acoustic environment is possible. The impact on patient outcomes needs to be evaluated.

**Supplementary Information:**

The online version contains supplementary material available at 10.1186/s13054-023-04685-2.

## Introduction

In recent years, there has been an unprecedented media coverage and visibility of critical care, mainly due to the COVID-19 pandemic [1]. While survival rates for patients admitted to intensive care units (ICUs) are continuously improving [2], the quality of survival is commonly suboptimal [3–5].

The interrelationship between the environment and health has been recognised since antiquity [6]. More recently, awareness that the physical environment affects one’s mood, behaviour, learning, cognitive function, general health, and sleep is growing [7–9]. However, the impact of the ICU environment on both morbidity and mortality has been vastly underestimated and hence under-investigated until recently.

Problems are complex: excessive noise, suboptimal lighting, social isolation, inability to personalise a bland environment, lack of views and access to nature, and lack of cognitive stimulation and distraction can all contribute to patients experiencing sleep deprivation, delirium, and mental health problems, leading to mortality and ongoing physical, cognitive, and psychological impairments after discharge [5, 7, 10, 11]. Family members have been reported to perceive the environment as threatening and stressful, contributing to psychological distress during and after a loved one’s ICU admission [12, 13]. Staff are also negatively affected by excessive noise and limited natural light and views. This can impact their physical and mental health, concentration, decision making, and contribute towards tension headaches and alarm fatigue [14, 15]. By contributing to patient confusion and delirium, the environment can increase risk of threats to staff safety through being exposed to verbal and physical aggression [16]. Current problems are likely to be exacerbated as more technologies are developed and deployed, enabling sicker patients to be admitted and survive an ICU admission, albeit with prolonged exposure to the toxic environment and subsequent higher incidence of delirium and other problems.

Information about the evidence used to shape ICU bedspace design over the years is sparse, with current designs varying greatly within and between countries [17]. In common with general hospital wards, ICUs were initially modelled on traditional open-plan Nightingale wards. With many early ICUs managed by anaesthetists [18], they were designed as an extension of the operating theatre to enable management of a heavily sedated patient. And while technology, care provision, and models of care in ICU have evolved rapidly over the years, with best practice now being the lightest sedation possible, the physical ICU environment has remained stagnant, not reflecting the dramatic changes in patient management that have occurred within their walls. The traditional environment is therefore no longer fit for purpose.

There are many complex reasons for this limited design innovation, including a previous lack of awareness of the negative impacts of the environment, costs associated with adding what are perceived as “extras” to a standardised design template, and feasibility of completing significant upgrades of existing ICUs. Moreover, frequently while harm of current practice is considered "acceptable" or "inevitable", potential harm from change is "unacceptable" and "avoidable" [19]. Importantly, current ICU designs and planning commonly focus on optimising clinical efficiencies; processes are led by bureaucrats, architects, and builders with limited opportunities for involvement for the end-users of the space—grassroot clinicians, patients, and their families [20], despite a growing recent awareness of the importance of consumer participation in health policy decision making and organisational change management processes [21, 22].

Optimising the environment has the potential for substantial positive impacts on patient outcomes and staff health and performance, thereby improving efficiency and effectiveness [23, 24]. There have been recent suggestions that biophilia, or connectivity with nature and green environments, should be incorporated into ICU designs [25, 26]. However, this may not always be possible to incorporate, especially in a retrofit solution. There is therefore a growing realisation that a significant redesign is required to address current problems. However, there is scant evidence upon which to create a ‘gold-standard’ ICU design and no agreement on what an ‘ideal’ ICU should achieve. An ‘ideal’ ICU design should reduce the incidence of preventable problems such as delirium, reduced sleep quantity and quality, loss of circadian rhythms, and ongoing problems after leaving ICU.

This manuscript describes the process of reconceptualising, designing, and retrofitting two innovative ICU bedspaces, and the early results of environmental upgrades.

## Methods

Mixed methods were used to gather and analyse data needed to meet the project aims. The interlinked aims of the project were to assess the existing environment and impact on all end-users, to identify problems and potential solutions, and to design and implement an optimised, evidence-based ICU bedspace. The project comprised several stages and linked sub-studies as depicted in Table 1.

**Table 1:** summary of project stages, activities, and outcomes. dBA = A-weighted decibel; RT = Reverberation Time

Stage	Activities and relevant studies	Outcomes
(1) Defining the problem	(1) Qualitative patient/family interviews (2) Qualitative staff interviews (3) Environmental studies (4) Literature review	(1) Draft list of patient-centred problems
(2) Designing solutions	(1) Co-design workshop (2) Extensive consultation and iteration (3) Creating solutions (4) Testing solutions	(1) Project requirements document—final list of requirements incorporated into the implementation plan
(3) Implementation planning	(1) Source implementation site/bedspaces (2) Investigation of selected bedspaces (3) Specific and prioritised list of recommendations produced (4) Architectural, design and technological plans developed (5) Stakeholder engagement process (6) Full-scale prototype built (7) Simulation training conducted	(1) Finalised architectural, design, and technological plans
(4) Building works	(1) Bedspaces isolated (2) Access and paths agreed on for building team and materials (3) Installation of agreed solutions (4) Finalising building works (5) Testing of lighting and acoustic environment	(1) Completion of two upgraded ICU bedspaces ready to accept patients
(5) Early evaluation	(1) Evaluation of light and acoustics	(1) Improved acoustic and lighting environments Reduced RT from 0.7 to 0.3 s Reduced monitor alarm volume by 8–11 dBA (as experienced by patients) Light that closely mimics natural light

### Stage 1: defining the problem

To ensure the project was fit for purpose for end-users, the initial step was to complete qualitative studies with patients, family members, and staff to understand the impact of the environment on patient experience and recovery, and potential solutions to perceived problems. A full description of the methods and results of these qualitative interviews have been previously published [20, 27]; a brief synopsis is provided below.

Seventeen patients, seven family members, and thirty ICU clinicians (medical, allied health, and nursing) were interviewed individually and in focus groups (staff only). Data were analysed using a framework approach [28]. Participating patients described ICU as scary and confronting, and highlighted issues such as noise and bright lights at night preventing sleep, while reporting that the ICU bedspaces were small and cluttered negatively affecting care provision. Other issues highlighted were an inability to personalise the environment to their needs and limited access to natural light/views, cognitive stimulation, and connectivity with family and the outside world [27]. Participating staff generally supported findings from the patient interviews and acknowledged that current bedspaces were suboptimal healing environments. They reported frustrations with their inabilities to personalise the environment, and highlighted how environmental features (e.g., noise and suboptimal lighting/lack of views) negatively impacted on staff health as well as ability to provide best care [20].

Next, various methods were used to objectively measure and evaluate the physical and sensory environment in the study ICU. Studies examined factors including light, sound, acoustics, and alarm frequency [29]. In parallel, a comprehensive literature review was completed to further develop a deep understanding of current problems linked with the ICU environment, potential solutions, and impact where any had been implemented and studied.

**Fig. 1:**
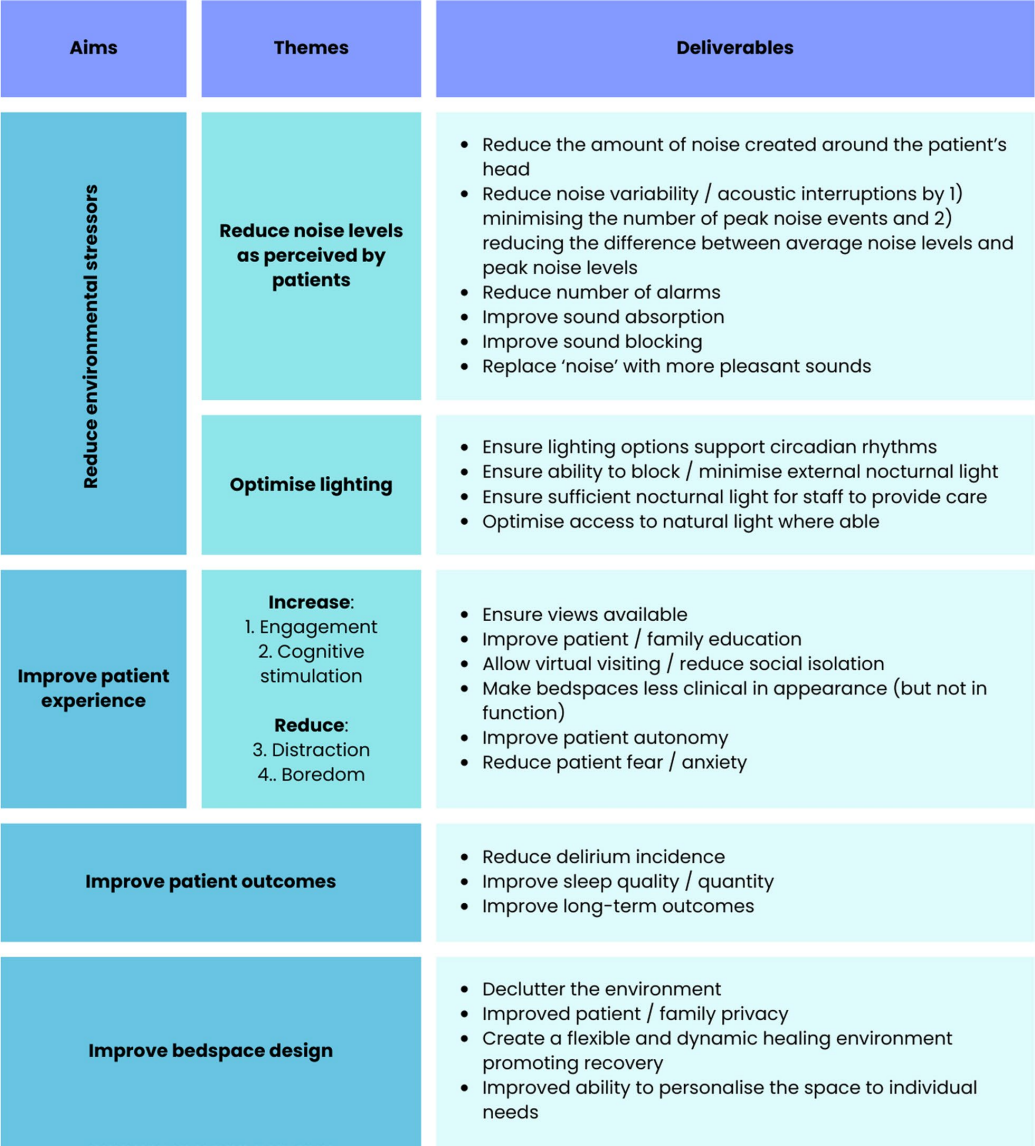
Initial draft list of patient-centred problems to be addressed for the project

The data collected from these interlinked sub-studies were then compiled into a draft list of patient-centred problems to be addressed for the project (Fig. 1).

### Stage 2: designing solutions

To address the multi-faceted problems identified in stage 1 and ensure the project continued to focus on the needs of all end-users of the bedspaces, a participatory design approach was employed to canvass and refine potential solutions. This was an iterative, interactive, and inclusive process involving all stakeholders (patients, families, staff, and industry partners) as project partners.

This phase commenced with a 2-day multidisciplinary stakeholder co-design workshop, with attendees including industry partners (builders, designers, architects, IT companies, and medical technology companies), clinicians, researchers, and former ICU patients (who shared their stories of admission to ICU and their life after ICU discharge). The workshop allowed conversations around potential solutions to commence from multiple angles. These included relevant software and IT improvements, technological innovations, and design and architectural solutions. Of interest, this was the first opportunity for any of the external collaborators to meet patients, despite having built or designed many hospitals previously.

**Fig. 2:**
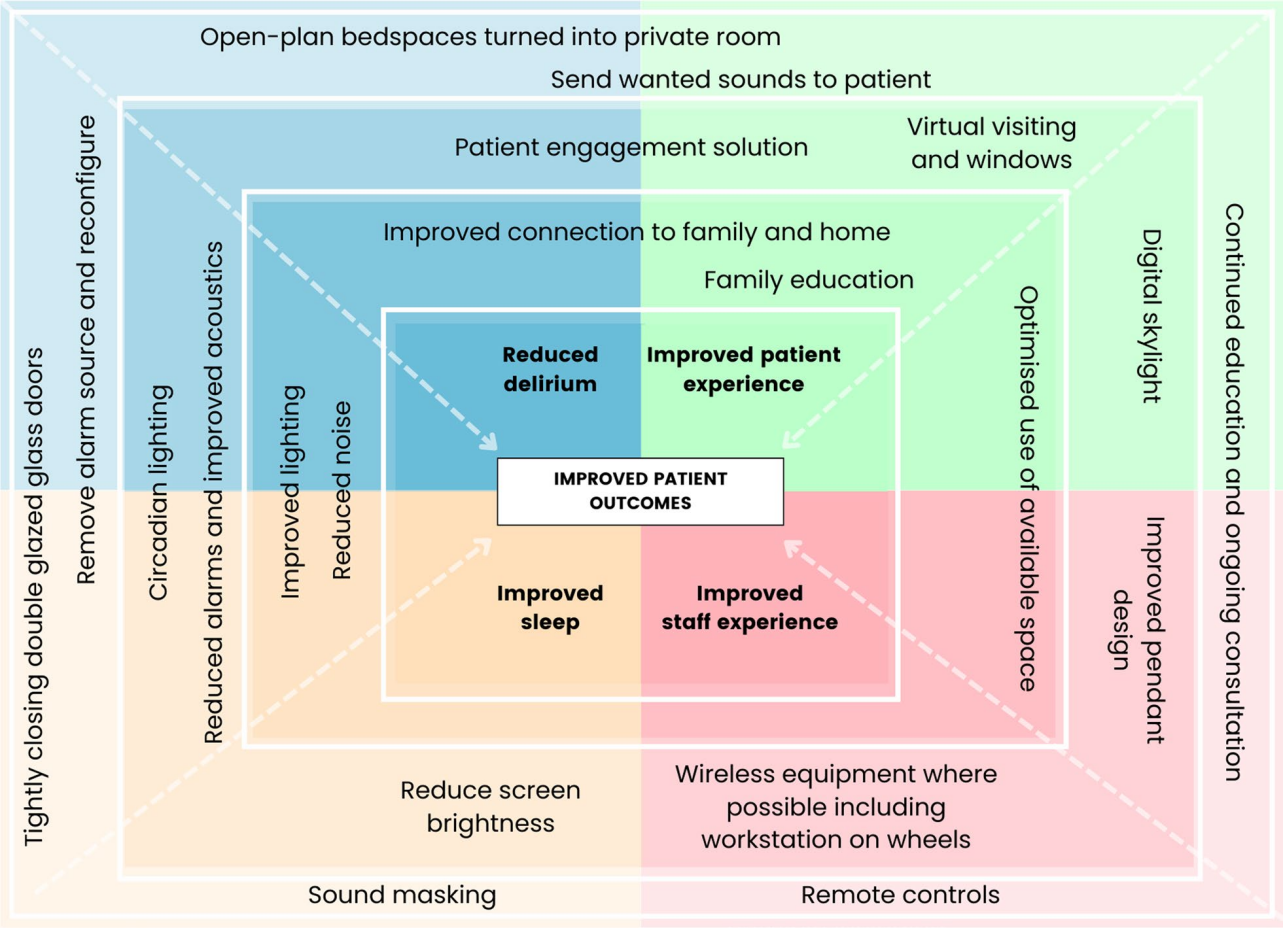
Simplified graphical description of the project action effect method diagram

Following the workshop, an agreed list of problems to be addressed was detailed in a draft project requirements document. This document had five streams (1: building and retrofitting, 2: materiality and acoustics, 3: clinical, 4: technology and integration, and 5: ‘other’) and outlined the requirements of an improved ICU environment (Additional file 1: Appendix 1). This document was finalised through extensive consultation and iteration, with all key stakeholders regularly meeting and engaged in suggesting and creating solutions, testing and finetuning them as relevant, before eventually agreeing on a final list of requirements to be incorporated into the proposed implementation plan. To support and guide this process, the action effect method was used to ensure all potential and suggested improvements contributed to the overall project aims (see Fig. 2 for a simplified graphical description and Additional file 2: Appendix 2 for the full diagram). This implementation and evaluation methodology uses a diagram to represent cause and effect relationships and is a commonly used framework to guide the implementation of complex quality improvement initiatives [30].

### Stage 3: implementation planning

After finalising requirements, a site suitable for implementation was identified. As each ICU varies regarding local context, patient population, and individual bedspace design, the solution for any given unit will always need to be individualised to the needs of that ICU and the population it serves. The project team engaged the clinical and management teams of our hospital to tailor plans for the complete upgrade of two ICU bedspaces according to the requirements identified in phases 1 and 2. This was planned to be a retrofit rebuild within a live ICU. Implementation was guided by the consolidated framework for implementation research (CFIR) innovation and implementation process domains [31].

**Fig. 3:**
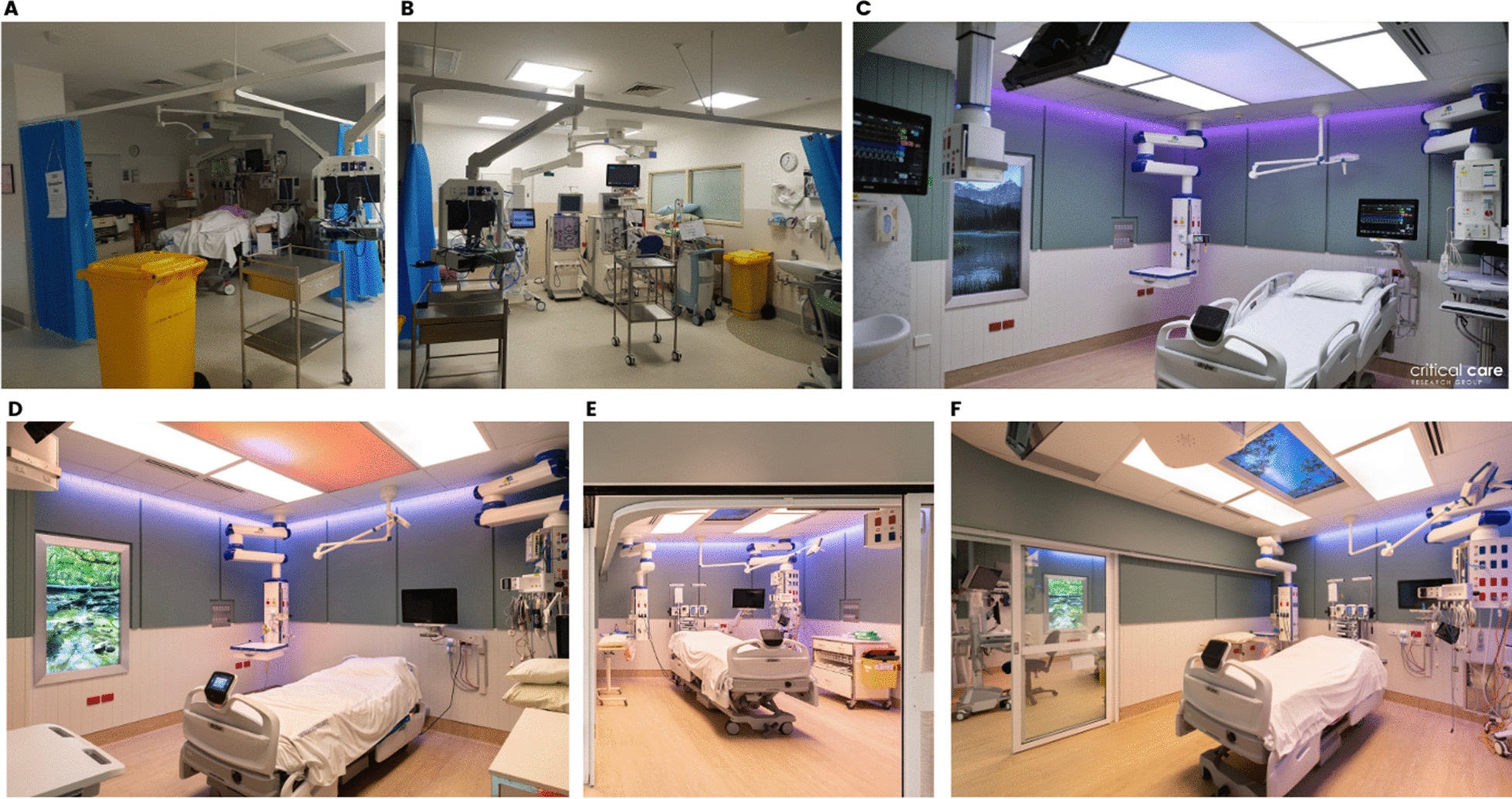
Bedspaces before (**A** and **B**) and after (**C**, **D**, **E**, and **F**) upgrade

The study ICU comprises 27 beds in three, nine-bed ‘pods’. Of these, 21 beds are open-plan and 6 are single rooms. Two internal and windowless open-plan ICU bedspaces (approximately 21 m^2^ each) were provided in a corner of one of the three ICU ‘pods’. Both were fully equipped ICU bedspaces, but previously used for simulation training and equipment storage (Fig. 3A and B). Following extensive consultation to ensure local context and priorities were fully understood, the two designated bedspaces were investigated, and a specific and prioritised list of recommendations for improvements produced. All recommendations were assessed against their impact on relevant patient outcomes (including impact on delirium, sleep, and experience) as defined in the requirements document and action effect diagram to ensure the main project aims were being met. The recommendations were also evaluated against the potential costs versus predicted benefit associated with implementation.

**Fig. 4:**
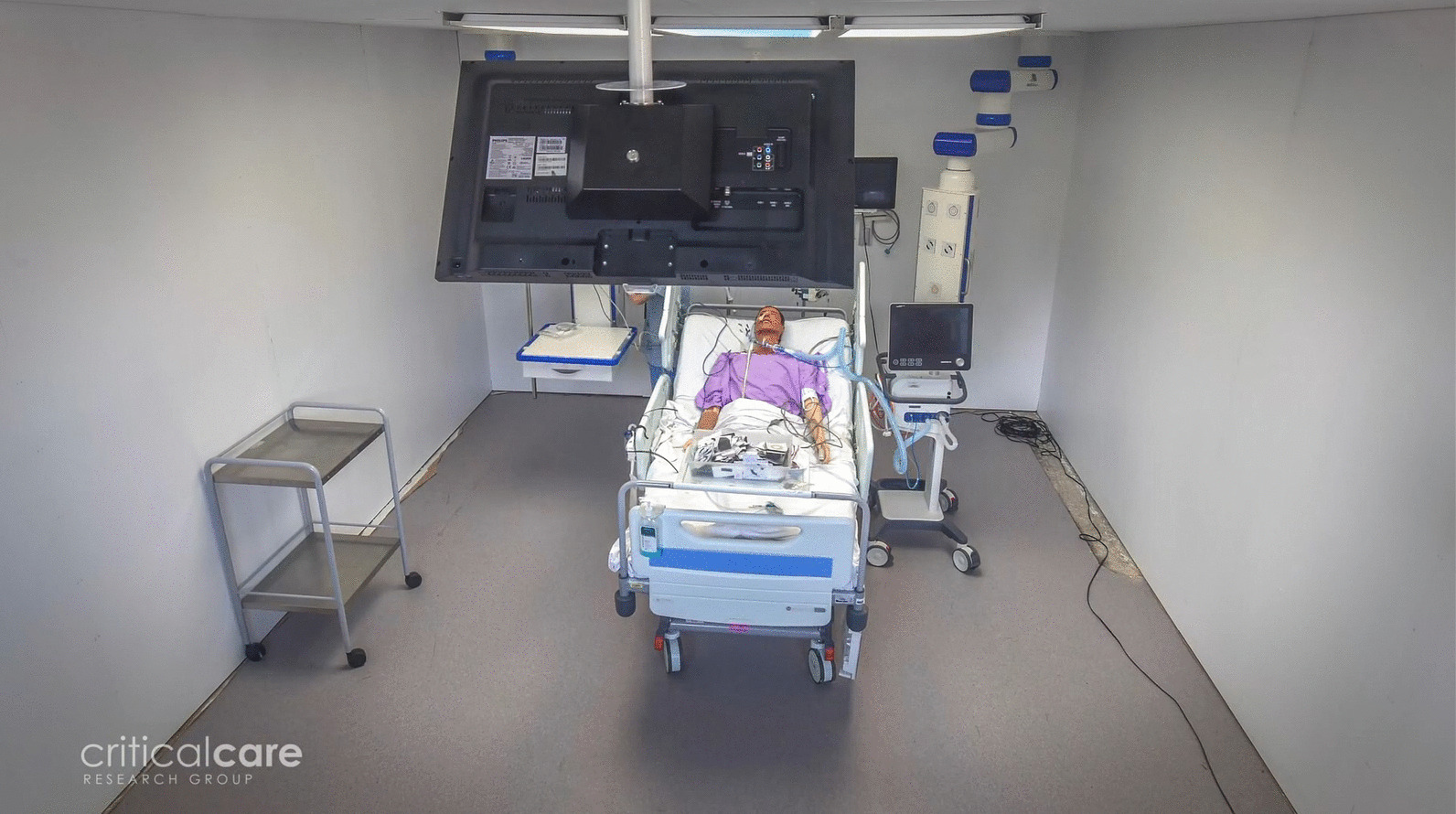
Prototype space built for staff consultation and simulation training

Early architectural, design, and technological plans were then developed followed by extensive stakeholder engagement conducted over 6 months to ensure all relevant stakeholders had an opportunity to provide feedback and help shape the final plans. Stakeholders involved included former patients and their families, ICU staff, building project managers, IT departments, biomedical technology services, hospital building and engineering services, infection control, external engineering companies, and relevant companies and contractors needed to provide, install, and integrate equipment and complete the final design. Plans were presented as they were updated, with feedback incorporated. Once plans were sufficiently progressed, a full-scale prototype (Fig. 4) was built in a separate building on the hospital campus, with proposed solutions installed and/or displayed. The prototype was used to gain practical feedback from staff who were able to view and physically test the proposed solutions and location of equipment through scheduled simulation training. Feedback was also sought from former patients and their families who were invited to visit the prototype. Feedback was incorporated as able, considering constraints and unmodifiable features associated with a retrofit solution in a live ICU, including existing infrastructure and walls, size constraints, and the location of the bedspaces. Finally, interior designers were consulted to develop colour and texture schemes. Plans were then finalised in preparation for the building works in the ICU.

## Results

### Stage 4: building works

Over a 10-week period, all building works were completed, and planned solutions were implemented and integrated with existing infrastructure where able (Fig. 3C–F). The ICU continued to operate as usual during this period, with the main impact being scheduled and communicated period of noise associated with the building works.

### Solutions implemented

Solutions were implemented as described below to address all problems identified in earlier project stages. Redundancy was included in both the electrical system and installed cabling to enable future changes and developments in response to improvements in technology and service requirements.

#### Noise

Noise levels were addressed in multiple ways.

### Sound absorption

**Fig. 5:**
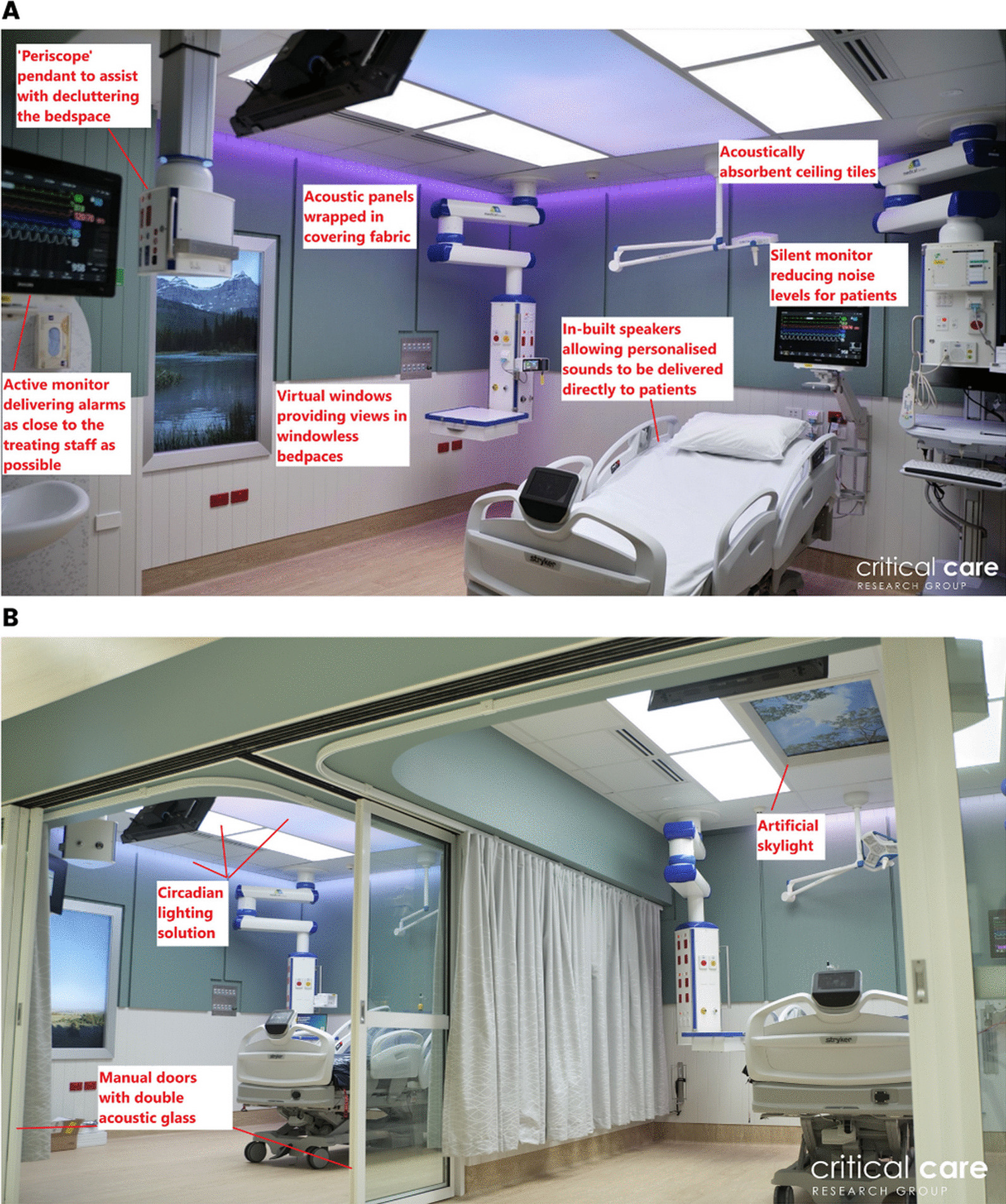
Features of the upgraded ICU bedspaces

Traditional hospital walls, ceiling, and floors are hard surfaces with limited sound absorption capability. Sound therefore reverberates and reflects within the bedspace, increasing the noise experienced by patients. Acoustically absorptive materials are freely available and used in other industries, however, most do not satisfy infection control criteria for hospital use, as they also absorb viruses and bacteria. A suitable wall covering fabric was identified that could be wrapped around the acoustic panel (noise reduction coefficient (NRC) 0.75), allowing sound to penetrate the fabric and be absorbed while resisting absorption of viruses and bacteria (Fig. 5A). The chosen product is also cleanable using standard hospital cleaning products, satisfying infection control criteria for use in ICU. A softer floor vinyl was chosen as well as acoustically absorbent ceiling tiles (NRC 0.21—Fig. 5A), all contributing to increasing the acoustic absorption, reducing reverberation, and reducing the reflection of any sound created within the bedspace.

## Sound blocking

To reduce externally created noise from surrounding bedspaces and clinical areas being transmitted into the two open-plan bedspaces, they were converted into single rooms. The bedspaces were separated by a wall (approximately 2/3), and a manual sliding door (approximately 1/3). At the front, manually operated doors were installed. These doors had a double layer of extra thick acoustic glass and optimal seals between the doors and the walls (Fig. 5B).

### Reduction of noise created within the bedspace and perception of created noise

Various strategies were used to reduce noise created within the bedspace and patients’ perception of created noise. These included repositioning alarms away from the top of the bed where able (Fig. 5A), reconfiguring alarm settings and other strategies to reduce the number of alarms, educating staff about the detrimental impact of excessive noise, and enabling staff to control and modify monitors from the nurses’ computer. Several strategies were also put in place to control or mask unavoidable noise being created, including sound masking and personalised music therapy.

#### Light

As these were internal and windowless bedspaces lacking natural daylight, optimising artificial light was critical. Ideally lighting should mimic natural daylight as closely as possible, be simple to use, and modifiable to meet individual patient requirements. Because no off-the-shelf solution satisfying these requirements was found, a bespoke solution was created (Fig. 5B). This provided a fully timed and programmed circadian lighting solution, as well as indirect illumination through peripheral lights reflecting on the walls. These peripheral lights can be modified, offering the opportunity to use different colours to change the feel of the room and help modify patients’ emotions.

Nocturnal settings were also programmed, providing a low level of illumination to support staff in providing necessary care activities safely and efficiently from a location unlikely to impact on patient’s sleep. Similarly, other nocturnal lighting solutions were also incorporated.

#### Patient connectivity, stimulation, distraction, and engagement

Static features, such as artworks, patterns, and decorative ceiling tiles, were considered to improve the patient experience. However, as patients’ have unique tastes and preferences regarding what they consider to be visually pleasing and beneficial, and these are static features that are difficult to change once implemented and impossible to personalise to individual needs, dynamic solutions were chosen instead. A patient entertainment system was incorporated, allowing various audio-visual content to be delivered. Other dynamic features such as an artificial skylight (Fig. 5B) and a virtual window (Fig. 5A), with several available videos that can be selected based on personal preferences, were therefore incorporated instead to provide a simulated experience of ‘outside’ and nature to the windowless bedspaces. Virtual visiting was enabled, allowing patients to communicate with family and friends. Materials and colours were carefully chosen, with colours specifically chosen to help reduce stress and pain [32, 33], and equipment installed outside of the line of sight of the patient as practicable, to make the space feel less clinical and overwhelming for the patient. Solutions such as a workstation on wheels and doors were included to improve patient and family privacy.

#### Solutions for staff

An updated nurse call was incorporated to enable the bedside nurse to communicate directly with other members of the ICU multidisciplinary team. The associated duress and emergency alarms were made accessible from multiple areas of the bedspace, improving staff safety.

To reduce perceived clutter and improve the ability for nursing staff to observe patients, several previously static features were made mobile. Modifications included the ability to view and control the patient monitor from a workstation on wheels, relocation of pendants such that they could be moved out of the way when not being used, and installation of a ‘periscope’ pendant (Fig. 5A) at the foot-end of the bed allowing gases and power to be supplied when needed but removed when not required.

### Stage 5: early evaluation

At the time of writing, evaluation is in the early stages and to date has focused on objective measures. An acoustic and lighting evaluation was performed immediately after the building works finished, but before patients were admitted to the bedspaces, ensuring there was time available to make necessary further modifications if the acoustic and lighting requirements had not been initially met.

A full description of the methods used to evaluate the optimised bedspace environment has been published [29], In brief, horizontal illuminance levels and spectral power distribution (SPD) were measured to evaluate the light intensity and wavelengths across the electromagnetic spectrum. Bedspace acoustics were tested via (1) background noise levels, (2) reverberation time (RT), and (3) acoustic privacy and separation between spaces. In addition, a Spartan™ Sound Level Meter was used to evaluate the change in sound levels as experienced by patients when moving monitor alarms away from the head of the bed. To test this, we placed the sound level meter next to the left ear of a simulation mannequin. We then compared alarms at all volumes (0–10) with the monitor situated 90 cm from the position of the patient’s head (simulating the normal monitor position) against the monitor situated 4.2 m from the position of the patient’s head (simulating sending alarms to the most common location of the bedside nurse and the approximate location of the active monitor in the upgraded bedspaces).

**Fig. 6:**
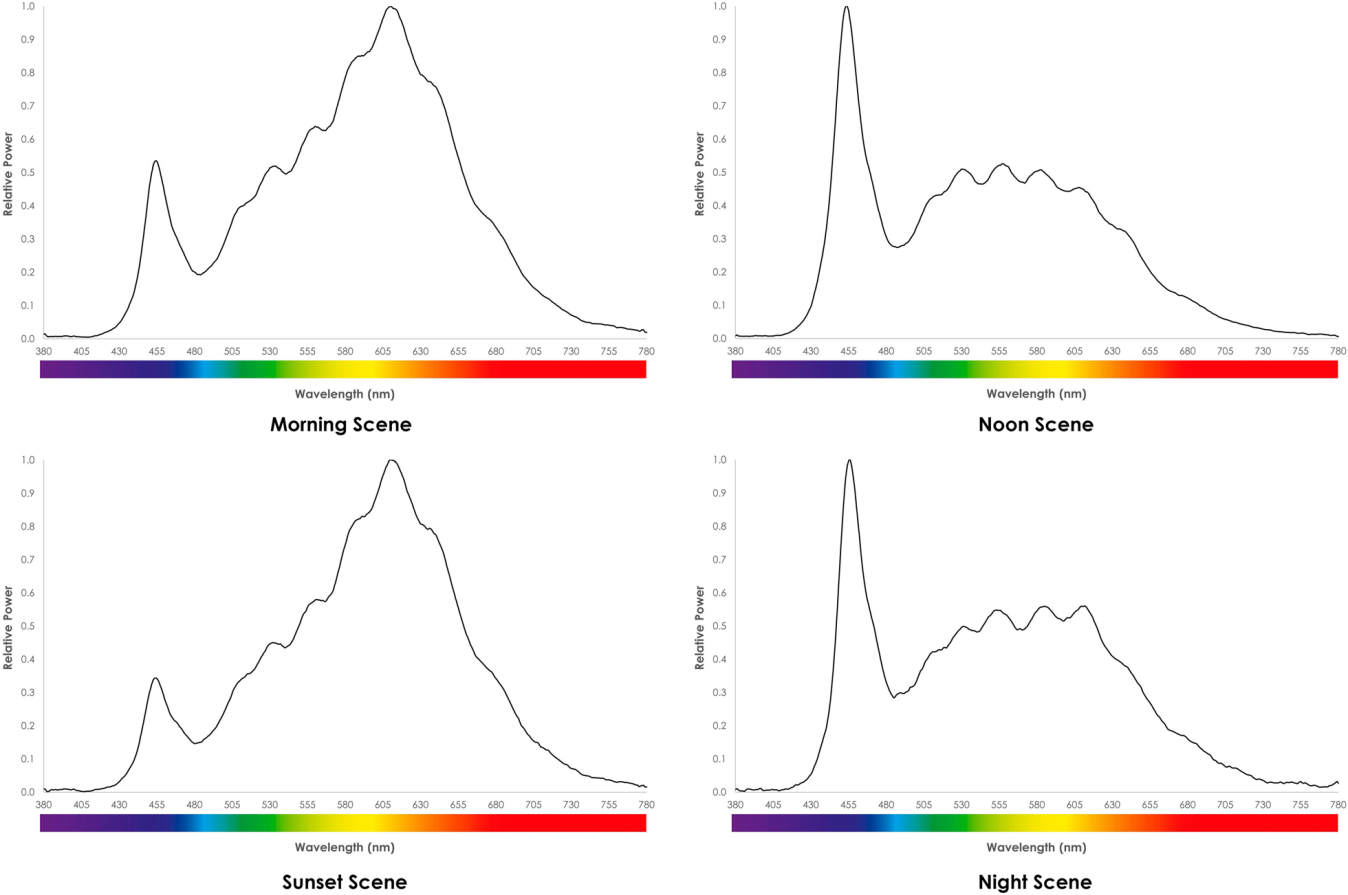
SPD in the upgraded bedspaces in the morning, noon, sunset, and at night

**Fig. 7:**
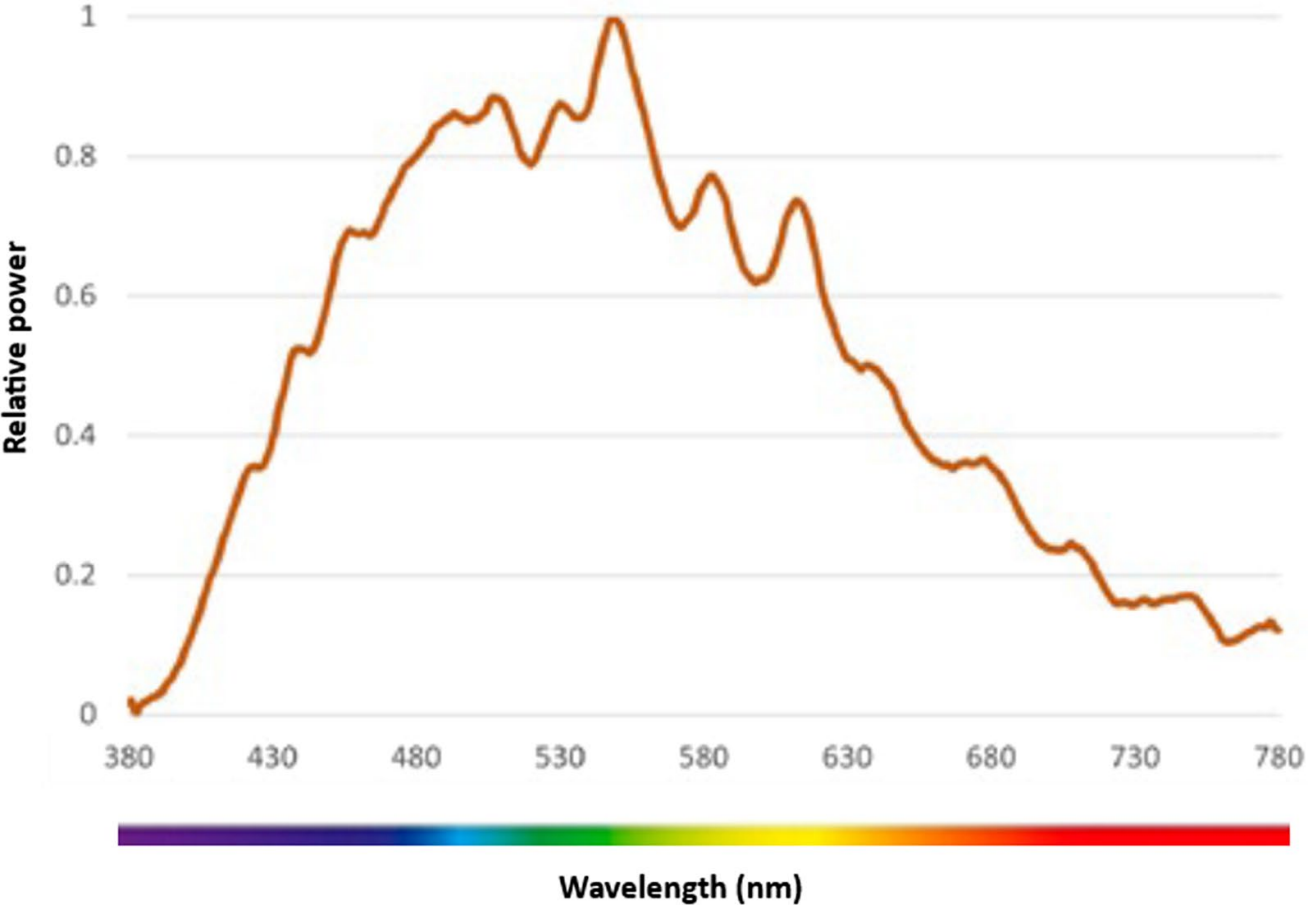
SPD in a bedspace with a window with all ceiling lights off (natural light only)

**Fig. 8:**
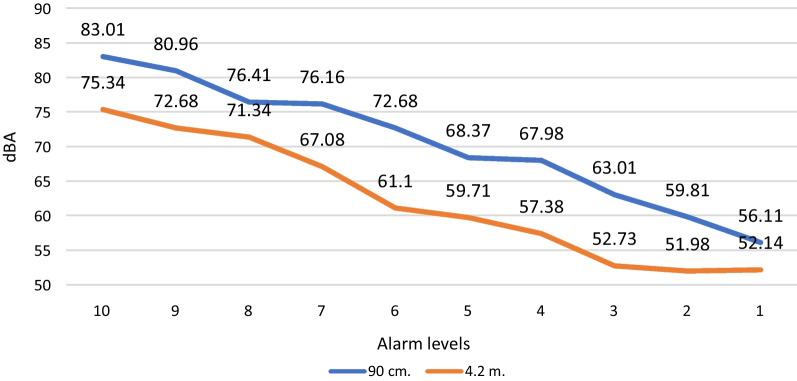
Summary of the maximum loudness (LAFMax—the maximum sound level with 'A' frequency weighting and fast time weighting) of monitor alarms as experienced by patients, comparing the traditional location of monitors against monitors being positioned close to the location of the bedside nurse. dBA = A-weighted decibel

A summary of the compounded SPD values in the upgraded bedspaces at various times during the day is presented in Fig. 6, compared with the SPD values in a bedspace with a window (all lights off—imitating effect of natural daylight) in Fig. 7. The RT in the upgraded bedspaces was 0.3 s (compared to 0.7 s before the rebuild, with recommended levels being less than 0.6 s) and the sound weighted level difference (Dw—measure of how much sound is blocked from entering the bedspace) was 21 dBA (A-weighted decibel—compared to approximately 0 dBA being blocked by the curtains at baseline). The background noise levels in the two bedspaces were 42 and 43dBA. The results from moving the monitor alarms further away from the patient’s head is shown in Fig. 8.

## Discussion

The improvements in ICU care and survival over the last 20 years are gratifying, but we must now look for quality not just quantity of survival. Despite growing awareness of the detrimental impact of the environment on patient outcomes, limited investment has been made in environmental redesign. This manuscript describes the process undertaken to improve the environment of two ICU bedspaces utilising best available and novel design principles and technology to address patient-centred problems common to most ICUs. Early outcomes demonstrate an improved acoustic and lighting environment, with reverberation time more than halved (demonstrating improved sound absorption within the bedspaces) as well as improved sound blocking from externally created sound, and a circadian lighting solution that closely mimic natural daylight compared to traditional electrical lighting. A separate manuscript has described the detailed lighting evaluation of the bedspaces before the implementation of the upgraded bedspaces, including a comparison between bedspaces with/without windows in different lighting conditions [29]. A key finding of that study was that in a windowed bedspace, the light available to patients closely mimicked natural light, but only when the ceiling lights were off. As soon as the ceiling lights were turned on, the light available to patients mimicked that of a windowless bedspace [29]. As can be seen in Figs. 6 and 7, the circadian lighting solution implemented in this project was found to closely mimic natural daylight, both with regards to the SPD (the ‘colour’ of the light) as well as the timing of delivery, delivering light very similar to the natural light we have evolved around and are dependent on for circadian rhythm entrainment. Moving the monitor alarms away from the patient’s head reduced the loudness of the alarms (as experienced by the patient) by between 8 and 11 dBA. A 10 dBA decrease equals approximately a halving of the perceived loudness of the noise and is the equivalent to the mean sound abatement achieved by earplugs [34].

Incorporating multiple partners and perspectives in a participatory design approach, especially the voices of the consumers, was essential for project success and enabled the project team to completely reconceptualise and future-proof the bedspaces, addressing real and identified problems experienced by ICU patients, family members, and staff locally and internationally. There has been a growing awareness of the importance of consumer involvement in various healthcare change management processes, but to date this does not seem to have translated into any meaningful involvement of the end-users when it comes to ICU bedspace design. Ensuring widespread collaboration and inclusion of all relevant stakeholders, rather than the traditional top-down approach, is essential for achieving effective healthcare innovation [35]. Although solutions were prioritised and chosen based on local needs of the study ICU, the problems addressed are universal and the process undertaken (and many of the solutions implemented) applicable and generalisable to ICUs (and other hospital wards) worldwide.

The negative impact of the environment on patient outcomes is likely to accelerate as new technology is introduced to sicker people. Noise levels are increasing, and there are more screens and other sources of nocturnal lights [36–38]. Sicker patients are admitted to ICU and surviving, but commonly requiring longer admissions, extending their time in contact with the environment [39]. And patients are more commonly managed with reduced sedation, providing them with opportunities to interact with the environment. However, the bland clinical environment provides nothing interesting or stimulating to interact with. Therefore, simple, unidimensional solutions aimed at masking the problems (such as ear plugs and eye masks) are not likely to be the optimal solutions, and efforts should be focussed on multicomponent solutions addressing the source of the problems. Similarly, current ICU design standards and guidelines are unlikely to meet the needs of ICU patients today and in the future and should be updated to reflect the importance of patient participation, ensuring that ICU bedspace designs considers the rapidly changing models of care and technologies available to create an efficient and safe working environment for staff and a healing environment for patients.

Recently, there have been several calls to ensure access to nature or green environments for patients in ICU [26]. Unfortunately, as this was a retrofit project with two windowless bedspaces, there was no feasible way of physically providing this. However, to address this important requirements, technological solutions were utilised to virtually provide this as best as possible. Virtual windows and skylights allow various dynamic sceneries with accompanying soundtracks to be displayed to patients. These can be individualised based on patients’ preferences. Similarly, nature videos and soundtracks/nature sounds were incorporated into the patient entertainment system, with the sounds delivered directly to the patients via wireless speakers built into the ICU beds. There is strong evidence linking contact with greenery to improved patient outcomes and staff health [26]. However, the effectiveness of digital solutions needs to be tested in future research.

Reducing noise and optimising lighting are obviously important aims, and this project has demonstrated that this is achievable. However, the ability to personalise the environment is also important. Every patient will have different needs of the environment and individual preferences. The ability to individualise parts of the environment such as the lighting (as an example) provides an opportunity to move the environment from being a passive cause of negative outcomes to potentially an active part of the care, where a ‘dose’ of the right environment at the right time can be used to optimise care provision. This also provides an amount of control and autonomy to the patient, something that is commonly lacking in traditional ICU designs.

This project has shown that environmental improvements are possible, albeit challenging, even as a retrofit in a live ICU, and that an improved lighting and acoustic environment can be created. Challenges faced during the implementation of this project included patient factors, staff factors, organisational factors, the retrofit nature of the implementation, technological limitations, and the fact that a pandemic started around the same time as the planning for project implementation. Many of these challenges (such as resistance to change when introducing new technology into an existing ICU) were expected, allowing proactive planning to occur. However, others were unexpected and required agility and ongoing open communication and dialogue with a diverse and large group of stakeholders.

Importantly, there is limited information available to determine whether environmental improvements make any difference to patient outcomes. Studies have reported that features such as circadian lighting solutions and physical barriers to reduce noise reduces the incidence of delirium and ICU length of stay [40]. Sleep has been shown to be improved by sound masking [41]. And as earplugs have been shown to reduce the incidence of delirium [42], it is likely that removing the noise in the first place or preventing it from disrupting patients’ sleep is likely to have the same effect. However, the authors have been unable to find any current literature on the impact of large environmental upgrades at this scale on patient outcomes. The next phase of our project will be to comprehensively evaluate project outcomes, both on patient experiences and outcomes, as well as on family members and staff. This evaluation will include qualitative interviews of patients, families, and staff members, and quantitative studies on patients’ sleep, circadian rhythms, delirium incidence, ICU outcomes, and physical/cognitive/psychological recovery 6-months after ICU discharge.

## Conclusion

Designing and implementing improved and patient-centred ICU bedspaces is feasible. More patient-friendly bedspaces will improve the patient and family experience without negatively impacting on staff’s ability to provide care. The impact on patient physical, cognitive, and psychological recovery while in ICU and after discharge is yet to be determined.

### Supplementary Information

**Additional file 1**: Project requirements document.

**Additional file 2**: Action effect method diagram.

## Data Availability

The datasets are available from the corresponding author on reasonable request.

## Abbreviations

CFIR: Consolidated framework for implementation research
dBA: A-weighted decibel
ICU: Intensive care unit
NRC: Noise reduction coefficient
RT: Reverberation time
SPD: Spectral power distribution
