# Supplementary material for: Creating the ICU of the future: patient-centred design to optimise recovery
Source: Crit Care. 2023 Oct 21;27:402. doi: 10.1186/s13054-023-04685-2 (PMC10589962; doi:10.1186/s13054-023-04685-2)
Supplement: Supplementary file 1 — Additional file 1: Project requirements document. [file 13054_2023_4685_MOESM1_ESM.pdf]

Requirement ID	Stream	Source Detail	Requirement
1	Building and retrofitting	Key requirements of the final product are that:	The solution shall be retrofittable.
2	Building and retrofitting	Key requirements of the final product are that:	The solution shall not reduce the number of ICU bedspaces available per unit.
3	Building and retrofitting	Key requirements of the final product are that:	The solution will improve the utilisations of current bedspaces / more efficient use of current space.
4	Building and retrofitting	Key requirements of the final product are that:	The solution shall not interfere with, or hinder access to, any ICU / monitoring equipment.
5	Building and retrofitting	Key requirements of the final product are that:	The solution must allow rapid access to all patients for a large team + equipment during emergencies.
6	Building and retrofitting	Key requirements of the final product are that:	The solution will have the ability to rapidly expand the available space around the patient.
7	Building and retrofitting	Key requirements of the final product are that:	The solution will accommodate the dynamic requirements of an ICU patient space, including all forms of patient transfers and exercise therapy.
8	Building and retrofitting	Key requirements of the final product are that:	The solution shall allow complete access to the head of the patient.
9	Building and retrofitting	Key requirements of the final product are that:	The solution will allow access to equipment and patient simultaneously.
10	Building and retrofitting	Key requirements of the final product are that:	Options to clinical pendants should be considered (but maintain the functionalities that pendants bring).
11	Building and retrofitting	Pendants	Numbers of clinical pendants required should be considered, and potentially minimised to 1 traditional pendant.
12	Building and retrofitting	Pendants	Clinical pendants will be optimised to allow maximum movement and flexibility.
13	Building and retrofitting	Pendants	Clinical pendants will be optimised to occupy the minimally required space / be as small as possible.
14	Building and retrofitting	Pendants	If the primary monitor is on a WOW – the head end monitor can be mounted on the wall in a location visible to staff rather than a pendant.
15	Building and retrofitting	Pendants	Periscope clinical pendants (wired) could be considered at the foot end of the bed (for ECMO etc.)
16	Building and retrofitting	Key requirements of the final product are that:	The solution will incorporate electrical cable reticulation.
17	Building and retrofitting	Key requirements of the final product are that:	The solution will incorporate data cable reticulation.
18	Building and retrofitting	Key requirements of the final product are that:	The solution will incorporate medical gas line reticulation.
19	Materiality & acoustics	Key requirements of the final product are that:	The solution shall adhere to all hygiene and infection control requirements.
20	Materiality & acoustics	Key requirements of the final product are that:	The solution will tolerate all ICU cleaning products (excluding Clinell)
21	Materiality & acoustics	Key requirements of the final product are that:	The solution will be easy to clean (noting non typical or clinical wall products are being potentially utilised within the space).
22	Materiality & acoustics	Key requirements of the final product are that:	The solution should utilise vacant real estate off the floor when able.
23	Materiality & acoustics	Key requirements of the final product are that:	The solution will ensure that anything suspended from the ceiling needs to be able to be moved completely out of the way
24	Materiality & acoustics	Key requirements of the final product are that:	The solution shall reduce the amount of noise produced by minimum 5 dB.
25	Materiality & acoustics	Key requirements of the final product are that:	The solution shall reduce the amount of perceived sound for the patient.
26	Materiality & acoustics	Reduce environmental stressors by:	Perceived sound will be reduced through sound masking.
27	Materiality & acoustics	Reduce environmental stressors by:	Sound masking can increase the background noise levels from 38 dB to 45-48 dB.
28	Materiality & acoustics	Reduce environmental stressors by:	Sound levels shall be reduced through optimal sound absorption.
29	Materiality & acoustics	Reduce environmental stressors by:	Sound levels shall be reduced through maximal sound blocking.
30	Materiality & acoustics	Reduce environmental stressors by:	Materials chosen for panels to achieve sound absorption and blocking will not reflect excessive light for patient comfort.
31	Materiality & acoustics	Reduce environmental stressors by:	Materials chosen for panels to achieve sound absorption and blocking will not allow excessive light for patient comfort to enter the bedspace.
32	Materiality & acoustics	Reduce environmental stressors by:	Maximum sound blocking will be achieved by extending the materials as close to the floor as possible.
33	Materiality & acoustics	Reduce environmental stressors by:	Sound produced by staff can be reduced through environmental monitoring with visual sound feedback.
34	Materiality & acoustics	Reduce environmental stressors by:	Sound produced by staff will be reduced through ongoing education.
35	Materiality & acoustics	Reduce environmental stressors by:	Sound produced shall be reduced through silent alarms / alarm management solutions / prioritising alarms.
36	Materiality & acoustics	Key requirements of the final product are that:	The solution shall reduce the amount of alarms produced by minimum 25%.
37	Materiality & acoustics	Reduce environmental stressors by:	Sound produced shall be reduced by including the ability to adjust the equipment remotely / from nurses computer.
38	Materiality & acoustics	Reduce environmental stressors by:	Sound produced will be reduced by ensuring bins close softly and quietly.
39	Materiality & acoustics	Reduce environmental stressors by:	Sound produced will be reduced by ensuring bins move more quietly.
40	Materiality & acoustics	Reduce environmental stressors by:	Sound produced will be reduced by ensuring the bins are placed in a location where they can be changed / emptied with minimum disturbance to the patient
41	Materiality & acoustics	Reduce environmental stressors by:	Sound produced shall be reduced by ensuring that all doors close quietly
42	Materiality & acoustics	Door closure	Sound produced will be reduced by ensuring that all doors are closed as the default position
43	Materiality & acoustics	Reduce environmental stressors by:	Sound produced can be reduced by ensuring there's a more gradual increase of water flow in the taps in the bedspace
44	Materiality & acoustics	Reduce environmental stressors by:	Sound produced will be reduced by ensuring appropriate impact protection in walls – e.g. aiming for RW 55
45	Materiality & acoustics	Reduce environmental stressors by:	Sound shall be delivered from the screen / PES directly to patient / speaker next to patient's head.
46	Materiality & acoustics	Reduce environmental stressors by:	Sound delivered from the screen / PES directly to patient / speaker next to patient's head shall be individually controlled / adjusted.
47	Materiality & acoustics	Reduce environmental stressors by:	The solution will be optimised for sleep.
48	Materiality & acoustics	Optimising the sleep environment by:	The solution will ensure darkness for patients at night.
49	Materiality & acoustics	Optimising the sleep environment by:	The solution shall miminise / remove light pollution at night.
50	Materiality & acoustics	Optimising the sleep environment by:	The solution will reduce light from monitors and displays at night.
51	Materiality & acoustics	Optimising the sleep environment by:	The solution will consider soft night light for staff at the computer station.
52	Materiality & acoustics	Optimising the sleep environment by:	The solution will consider soft night light for staff to allow completion of clinical duties and observations, without disturbing the patient.
53	Materiality & acoustics	Optimising the sleep environment by:	The solution considers soft, soundless and backlit computer keyboards or soft night light for staff visibility
54	Materiality & acoustics	Optimising the sleep environment by:	The solution considers the ability to modify bedspace temperature and individualise / diurnal temperature variations.
55	Materiality & acoustics	Optimising the sleep environment by:	The solution will reduce the need for nocturnal disturbance and care activities.
56	Materiality & acoustics	Optimising the sleep environment by:	The solution shall provide optimal acoustics.
57	Materiality & acoustics	Optimising the sleep environment by:	The solution shall provide a quiet nocturnal space.
58	Materiality & acoustics	Optimising the sleep environment by:	Biomedical device monitoring operates wirelessly whenever possible, including ECG, and taking into consideration quantity of wirelessly connected devices
59	Materiality & acoustics	Optimising the sleep environment by:	The solution will incorporate measurement of patient sleep through suitable solutions.
60	Materiality & acoustics	Optimising the sleep environment by:	The solution incorporates measurement of patient sleep through patient feedback and PREM + reports / feedback to treating clinicians.
61	Clinical	Product	The solution will support optimisation of work processes / workflow for staff and make care delivery more efficient.
62	Clinical	Product	The solution will allow equipment and consumables to be easily accessible to staff to enable better workflow.
63	Clinical	Product	The solution incorporates a bigger workbench / surface if possible
64	Clinical	Workbench	The workbench / surface is potentially integrated into the wall
65	Clinical	Workbench	The workbench / surface is height adjustible if possible
66	Clinical	Workbench	The workbench / surface has room for trolleys underneath to reduce clutter – including lockable medication drawer
67	Clinical	Product	The solution will be ergonomic and safe for staff to use.
68	Clinical	Ergonomics	The height of the shorter staff members will be considered when things are placed on the walls

69	Clinical	Product	The solution maintains or decreases current risk of staff injuries.
70	Clinical	Product	The solution reduces the risk of patient injury.
71	Clinical	Product	The solution is intuitive, easy to use and learn for staff.
72	Clinical	Family Participation	The solution improves active family participation.
73	Clinical	Family Participation	The solution will improve how patients and their family are engaged with care, and facilitate active involvement.
74	Clinical	Family Participation	The solution shall improve the ability for the patient to connect with family and friends by allowing access to virtual visiting
75	Clinical	Family Participation	The solution shall improve the ability for the patient to connect with family and friends through a multi-purpose screen / PES.
76	Clinical	Family Participation	The solution will investigate a suitable space for family members and other visitors to feel comfortable without getting in the way of clinical care activities
77	Clinical	Family Participation	This space could include a pull down desk (or something similar) to enable visitors working on a computer etc.
78	Clinical	Family Participation	This space could include a comfortable chair that can be reclined and allow sleep in situations where family members are allowed to stay overnight
79	Clinical	Environment	The solution shall reduce environmental stressors.
80	Clinical	Environment	The solution shall incorporate diurnal lighting.
81	Clinical	Environment	Diurnal lighting will be able to be controlled and adjusted.
82	Clinical	Environment	Diurnal lighting will be able to be individually controlled and adjusted.
83	Clinical	Environment	The solution shall improve the ability to access natural light / face the window (if room is windowed).
84	Clinical	Environment	The solution shall provide a flexible space that has the ability to be open or closed.
85	Clinical	Environment	The front door (or relevant solution to close the space) will allow visualisation of patient from outside the bedspace
86	Clinical	Environment	If glass is used for this, consider using thicker standard glass rather than acoustic glass (if acoustic blocking / absorption is adequate)
87	Clinical	Personalised space	Voice activation / intercom could be used to allow patient privacy (for appropriate patients).
88	Clinical	Cognitive Stimulation	The solution shall improve cognitive and mental stimulation.
89	Clinical	Cognitive Stimulation	The solution shall improve access to distractions such as TV, internet, movies, radio, games, nature movies.
90	Clinical	Cultural Awareness	The solution shall provide access to indigenous artwork, entertainment & visuals etc.
91	Clinical	Patient Journey	The solution will improve and facilitate the journey back to normalcy.
92	Clinical	Patient Journey	The solution may improve the transition of a patient from ICU to wards.
93	Clinical	Patient Autonomy	The solution shall allow a patient to individualise and modify aspects of the space incl. light, sound, environment, temperature, music, views, space etc.
94	Clinical	Patient Autonomy	The solution shall give the patient improved control, autonomy and independence, e.g. through PES and nurse call system
95	Clinical	Patient Journey	The solution will improve the ability of all staff members to provide care for the patient, regarding design of the space and technology integration
96	Clinical	Patient Journey	The solution shall decrease clutter and unnecessary equipment.
97	Clinical	Patient Journey	The solution will decrease the amount of leads and cables where possible.
98	Clinical	Patient Journey	The patient and their current condition can be seen from a central area.
99	Clinical	Patient Journey	The patient and their current condition can be seen from a central area on a screen.
100	Clinical	Patient Journey	Staff shall be able to simultaneously view and look after two patients at once (not necessarily next to each other), through patient monitoring system.
101	Clinical	Patient Journey	The solution shall ensure easy ingress / egress for the patient.
102	Clinical	Patient Journey	The solution shall ensure easy ingress / egress for equipment.
103	Clinical	Patient Journey	The solution shall ensure easy ingress / egress for staff.
104	Clinical	Patient Journey	The solution will ensure easy and rapid access to all equipment and consumables.
105	Clinical	Patient Journey	The solution shall ensure easy and rapid access to the head of the bed / patient.
106	Clinical	Patient Journey	The solution improves staff efficiencies by reducing time spent on finding / moving equipment.
107	Clinical	Patient Journey	The solution will reduce the incidence of ICU delirium, post ICU cognitive and mental complications.
108	Clinical	Delirium	The solution will reduce the impact of delirium.
109	Clinical	Delirium	The solution will help re-orientate the patient.
110	Clinical	Delirium	The solution will help re-orientate the patient by being able to view real-time streaming of the outside world / from TPCH cameras, viewable on thte PES.
111	Clinical	Patient Journey	The solution shall improve the patient's journey through ICU.
112	Clinical	Patient Journey	The solution allows patients and families to provide feedback throughout their journey.
113	Technology & Integration	General	The solution shall have the capacity to incorporate and adapt to future changes in technology and service requirements.
114	Technology & Integration	General	Mobile solutions will be used wherever possible
115	Technology & Integration	Duress alarms	The solution shall incorporate easy and multiple access to nurse call / staff duress alarms from key areas of the bedspace.
116	Technology & Integration	Duress alarms	Staff duress alarms could be voice activated.
117	Technology & Integration	Environment	The solution will incorporate sensors for continuous environmental monitoring, including sound, light, and temperature.
118	Technology & Integration	Environment	The sensors will be able to produce meaningful clinical reports + send alerts in real-time to caregiver when environmental parameters falls outside pre-determined levels.
119	Technology & Integration	Audio-Visual	The solution shall incorporate a multi-purpose screen for patients.
120	Technology & Integration	Audio-Visual	The patient screen might be a large tablet device with all required functionalities (rather than PES / TVs) but a larger fixed screen preferable.
121	Technology & Integration	Audio-Visual	If used, tablet devices should be multi-use if possible (e.g. ability for staff to use if bed unoccupied).
122	Technology & Integration	Audio-Visual	The patient screen shall be able to display free to air TV, relevant BYO pay TV services, skype / virtual visiting, Spotify / music streaming services, nature scenes (with accompanying sounds), news feeds, local weather forecast, daily timetable, time and date etc.
123	Technology & Integration	Audio-Visual	The patient screen may be able to display relevant information from the daily plan / relevant handover information to inform the patient / family member.
124	Technology & Integration	Audio-Visual	The patient screen could connect to the guest network (or something similar) rather than hospital network.
125	Technology & Integration	Audio-Visual	The patient screen should have an interface for patients with limited upper limb mobility unable to use a tablet.
126	Technology & Integration	Audio-Visual	The solution will support the display of dashboard / information on a screen.
127	Technology & Integration	Education & Awareness	The solution shall support better ICU awareness and education for patients & their families.
128	Technology & Integration	Education & Awareness	The education app / delivery method shall facilitate improved patient education about their condition and what's happening to them.
129	Technology & Integration	Education & Awareness	The education app / delivery method may be accessible prior to admission for elective patients.
130	Technology & Integration	Education & Awareness	The education app / delivery method shall ensure information and the environment is personalised by allowing patients / family members to input patient likes etc.
131	Technology & Integration	Education & Awareness	Patients / family members will be able to input patient likes etc into the education app / delivery method prior to admission or as early as possible post admission.
132	Technology & Integration	Education & Awareness	The education app / delivery method may be accessible for family members no matter where they are (e.g. app available from home).

133	Technology & Integration	Education & Awareness	The education app / delivery method shall facilitate improved family (including children) education and preparation that reduces how intimidating they find ICU.
134	Technology & Integration	Education & Awareness	The solution may incorporate a tablet device available at the bedside.
135	Technology & Integration	Education & Awareness	The education app will operate on a tablet device.
136	Technology & Integration	Education & Awareness	The solution may incorporate a tablet device that integrates / communicates with relevant technology / screens etc.
137	Technology & Integration	Patient Engagement	The patient interface / tablet will allow data input and output.
138	Technology & Integration	Patient Engagement	The patient interface / tablet may be capable of monitoring the environment.
139	Technology & Integration	Patient Engagement	The patient interface / tablet may be capable of review, analysis, and modification of environmental aspects such as light and sound.
140	Technology & Integration	Patient Engagement	The patient interface / tablet will allow patient and family feedback to be captured.
141	Technology & Integration	Patient Engagement	The patient interface / tablet will allow PREMs to be captured.
142	Technology & Integration	Patient Engagement	The patient interface / tablet will provide patient goal setting.
143	Technology & Integration	Patient Engagement	The patient interface / tablet may provide a "Menu" for family to assist with care.
144	Technology & Integration	Patient Engagement	The patient interface / tablet may send requests to staff for action.
145	Technology & Integration	Patient Engagement	The patient interface / tablet may provide analytics to continuously evaluate what is being used.
146	Technology & Integration	Patient Engagement	The patient interface / tablet will incorporate a patient diary.
147	Technology & Integration	Patient Engagement	The patient interface / tablet will incorporate a delirium screening app.
148	Technology & Integration	Patient Engagement	The delirium screening app will have the ability to produce reports to clinicians.
149	Technology & Integration	Patient Engagement	The solution will allow the vast majority of patients to interact with the environment.
150	Technology & Integration	Patient Engagement	The solution will consider patients with limited upper limb movement & communication.
151	Technology & Integration	Patient Engagement	The solution shall incorporate customisable, music streaming services.
152	Technology & Integration	General	Technology shall allow the space to be indvidualised based on patient's specific needs & requirements.
153	Technology & Integration	Data	The solution will interface with existing technology / IT solutions such as Metavision and IEMR.
154	Technology & Integration	Interfacing	The solution should minimise the amount of secure internet / network access where possible.
155	Technology & Integration	Interfacing	All equipment will be integrated and interfaced as able.
156	Technology & Integration	Clinical	Radiation exposure and levels / EMC in the new bed-space shall be within safe levels.
157	Technology & Integration	Wireless	Wireless network will allow VLAN segregation of biomedical devices.
158	Technology & Integration	Wireless	Wireless solutions will not interfere with each other i.e. avoid co-channel interference.
159	Technology & Integration	Workstation on wheels	The nursing computer shall be mobile (Workstation on Wheels (WOW)), and shall be able to be moved to various positions to help facilitate patients facing in different directions.
160	Technology & Integration	Workstation on wheels	The WOW may need to accommodate dual screens potentially (1 for monitoring / clinical data, 1 for metavision / data input).
161	Technology & Integration	Workstation on wheels	The WOW shall be fully mobile, and able to move to all parts of the bedspace + outside bedspace if required.
162	Technology & Integration	Workstation on wheels	The WOW will have a minimum of 6 hour battery charge.
163	Technology & Integration	Workstation on wheels	Charging stations for the WOW will be incorporated in, or near, the bedspace.
164	Technology & Integration	Workstation on wheels	Charging points for WOW could be wireless if possible (induction).
165	Technology & Integration	Workstation on wheels	The WOW will be flexible to allow for future upgrades and changes - e.g. change to tablet devices etc.
166	Technology & Integration	Wired Network	The solution will include backup network ports on both sides of the bed.
167	Technology & Integration	The new prototype will:	The solution shall incorporate technology that supports a better experience for patients and their loved ones.
168	Technology & Integration	Design of ICT solutions must consider the patient first and strive to improve their experience:	ICT technology will deliver integrated rather than proprietary solutions.
169	Technology & Integration	Design of ICT solutions must consider the patient first and strive to improve their experience:	ICT technology solutions will put the patient at the forefront that support high levels of engagement in their treatment, care, recovery and rehabilitation processes.
170	Technology & Integration	Design of ICT solutions must consider the patient first and strive to improve their experience:	ICT technology solutions will not inhibit interaction between the patient and those providing care or other services.
171	Technology & Integration	Clinician engagement in the selection and design of ICT for the new prototype is essential, to ensure that these systems are fit for purpose and support efficient clinical processes:	ICT solutions will be easy to use, integrated and always reliable.
172	Technology & Integration	Clinician engagement in the selection and design of ICT for the new prototype is essential, to ensure that these systems are fit for purpose and support efficient clinical processes:	Systems will be integrated wherever possible.
173	Technology & Integration	Solutions should have capacity to allow for future change, growth and progressive extension:	ICT infrastructure will be able to be replaced / upgraded without the need for destructive works.
174	Technology & Integration	Solutions should have capacity to allow for future change, growth and progressive extension:	Solutions will be scalable and flexible.
175	Technology & Integration	Solutions should have capacity to allow for future change, growth and progressive extension:	Solutions will use products that can be integrated, and are based on industry standards.
176	Technology & Integration	Technologies must operate within the broader ICT environment of Qld Health and MNHHS. Therefore, adherence to agreed standards is essential.	Solutions that connect to the ICT network will comply with relevant ICT Standards.
177	Technology & Integration	The new prototype will:	The solution can support the use of RTLS technology.
178	Technology & Integration	The solution should also be capable of integrating with a number of other systems to provide the following functionality:	The solution may indicate staff presence via the RTLS solution that displays staff names on the screen when entering the room.
179	Technology & Integration	The bedspace will require a robust, highly available Medical Grade Network.	The network shall be capable of supporting multiple medical equipment devices.
180	Technology & Integration	The bedspace will require a robust, highly available Medical Grade Network.	The network shall be capable of supporting existing medical equipment devices.
181	Technology & Integration	The bedspace will require a robust, highly available Medical Grade Network.	The network shall be capable of supporting multiple vendor solutions.
182	Technology & Integration	The bedspace will require a robust, highly available Medical Grade Network.	The network will be continuously monitored by an enterprise grade network monitoring solution.
183	Technology & Integration	The bedspace will require a robust, highly available Medical Grade Network.	The network will be designed with consideration for the IEC80001-2-3: Application of Risk Management for IT networks incorporating medical devices - Part 2-3: Guidance for wireless networks.
184	Technology & Integration	The bedspace will require a robust, highly available Medical Grade Network.	The wireless network will have seamless connectivity.
185	Technology & Integration	The bedspace will require a robust, highly available Medical Grade Network.	The wireless network will be high-grade in the two bed-spaces being updated at TPCH and have complete room coverage as demonstrated by wireless heatmap.
186	Technology & Integration	The bedspace will require a robust, highly available Medical Grade Network.	The solution will minimise how often it touches the patient / QH network and utilise the guest network whenever possible.
187	Technology & Integration	The bedspace will require a robust, highly available Medical Grade Network.	Wireless Biomedical Device connectivity should be via the ICT converged network.
188	Technology & Integration	The bedspace will require a robust, highly available Medical Grade Network.	Wireless Biomedical Device connectivity will cater for appropriate levels of security to protect data and devices from malicious activity.
189	Technology & Integration	Adequate planning for UPS and emergency power supplies is critical.	Critical ICT systems & networks will be connected to emergency power feeds, including UPS for those systems that are unable to handle a loss of power.
190	Technology & Integration	Software design	Devices and equipment will communicate and integrate within bedspace.
191	Technology & Integration	Software design	Relevant data will communicate with hub / computer external to bedspace (e.g. nurses station).

192	Technology & Integration	Software design	Applications operating on PCs/tablets etc will be able to be remotely deployed.
193	Technology & Integration	Software design	Applications operating on PCs/tablets etc will be able to be remotely deployed by Microsoft System Installer (MSI).
194	Technology & Integration	Biomedical device - Alarm management	Biomedical devices can send inputs and alarm messages (alerts) to fixed devices (i.e. computers, WOWs) whenever possible.
195	Technology & Integration	Biomedical device - Alarm management	Biomedical devices can send inputs and alarm messages (alerts) to portable devices (i.e. smart phones / wireless handsets) whenever possible.
196	Technology & Integration	Biomedical device - Alarm management	Biomedical devices can send inputs and alarm messages (alerts) to a central console / monitor / workstation.
197	Technology & Integration	Biomedical device - Alarm management	Patient monitors next to patients will be configured to operate silently.
198	Technology & Integration	Biomedical device - Alarm management	Patient monitors may be configured with threshholds that trigger an audible alert when operating silently.
199	Technology & Integration	Biomedical device - Alarm management	Staff may manually trigger an audible alert on a patient monitor that is operating silently.
200	Technology & Integration	Biomedical device - Alarm management	Patient ventilators may be configured to operate silently if possible.
201	Technology & Integration	Biomedical device - Alarm management	Patient ventilators may be configured with threshholds that trigger an audible alert when operating silently.
202	Technology & Integration	Biomedical device - Alarm management	Staff may manually trigger an audible alert on a patient ventilator that is operating silently.
203	Technology & Integration	Server	The solution will support rack-based services.
204	Technology & Integration	Server	The solution will support cloud-based services.
205	Technology & Integration	Server	The solution will support server virtualisation.
206	Technology & Integration	Server	The solution will preference server virtualisation over rack-based services.
207	Technology & Integration	Network Connectivity	The solution will preference dynamic network connectivity, as opposed to fixed IP-based connectivity.
208	Technology & Integration	Network Connectivity	The solution will operate across standard TCP/IP network using IPv4 network protocol.
209	Technology & Integration	Network Connectivity	The solution will support H.320/H.323 network protocol for video-conferencing.
210	Technology & Integration	Remote Access	The solution will not connect directly to the Internet.
211	Technology & Integration	Remote Access	The solution may be remotely supported if required.
212	Technology & Integration	Security	The solution will operate securely in the network environment.
213	Technology & Integration	Security	The solution hardware and software will be capable of inter-operating with anti-virus software.
214	Technology & Integration	Security	The solution will allow authentication services to authorise the hardware / software to operate in the network environment.
215	Technology & Integration	Security	The solution hardware / software will allow patches / revisions to be applied within the live network environment.
216	Project principle	Key requirements of the final product are to:	The solution shall be cost-effective over a 10-year period.
217	Project principle	Key requirements of the final product are to:	The solution shall not increase the operating costs of the ICU.
218	Project principle	Key requirements of the final product are to:	Solutions will be selected having considered both implementation costs and recurrent costs.
219	Other	Change management	Robust change management principles will be applied in preparation for implementation of the solution.
220	Other	Change management	Staff will be able to continuously provide feedback.
221	Other	Change management	Simulation scenarios will be available for staff training
222	Other	Change management	Staff training will be provided in a timely manner.
223	Other	Research & quality assurance	Relevant data will be easily downloaded.
