# Supplementary figures and images for: Creating the ICU of the future: patient-centred design to optimise recovery
Source: Crit Care. 2023 Oct 21;27:402. doi: 10.1186/s13054-023-04685-2 (PMC10589962; doi:10.1186/s13054-023-04685-2)

## Slide 1
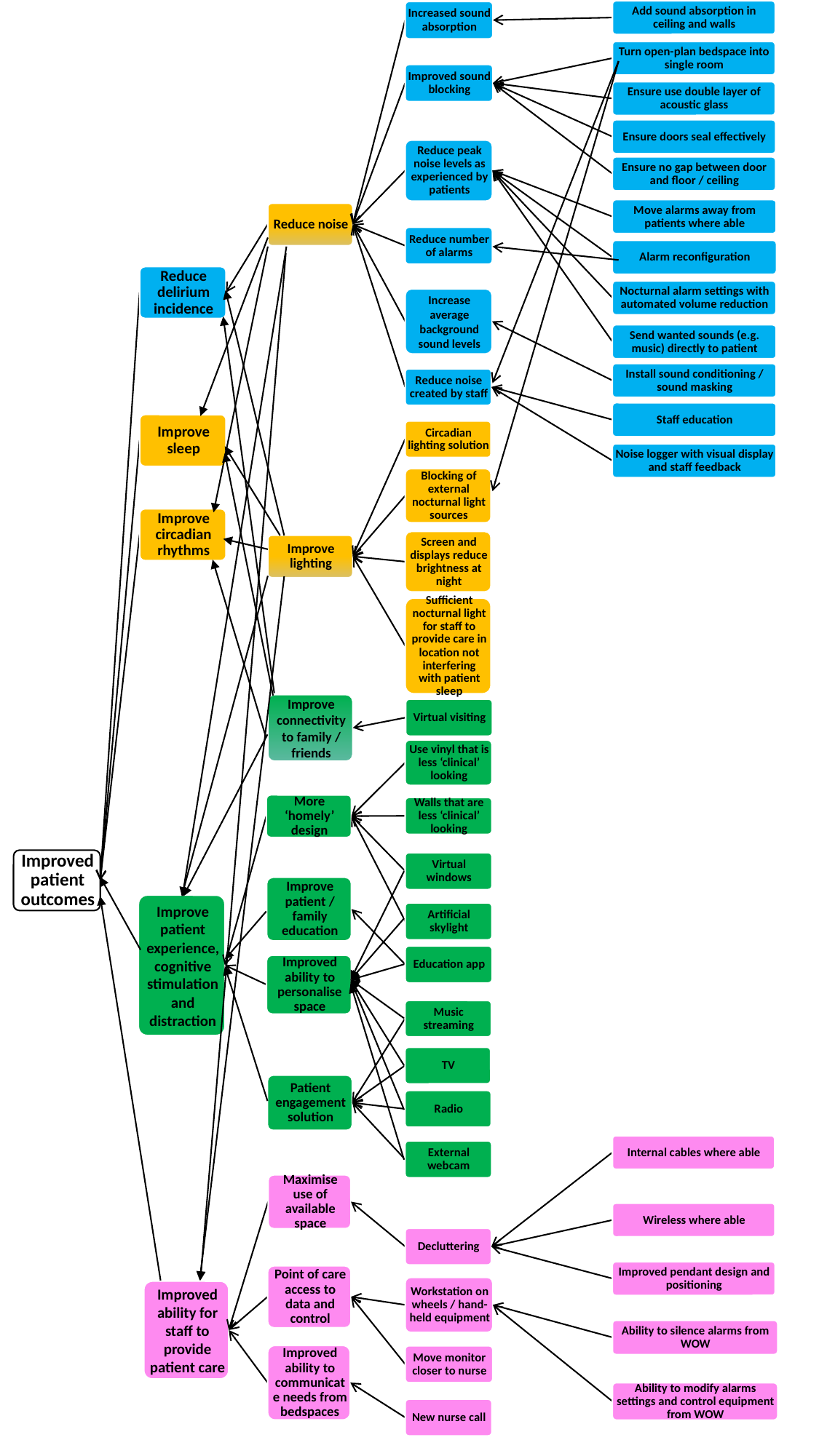

Supplement: Supplementary file 2 — Additional file 2: Action effect method diagram. [file 13054_2023_4685_MOESM2_ESM.pptx]
